# Transcatheter Versus Surgical Valve Repair in Patients with Severe Mitral Regurgitation

**DOI:** 10.3390/jpm12010090

**Published:** 2022-01-11

**Authors:** Matthias Koschutnik, Varius Dannenberg, Carolina Donà, Christian Nitsche, Andreas A. Kammerlander, Sophia Koschatko, Daniel Zimpfer, Martin Hülsmann, Stefan Aschauer, Matthias Schneider, Philipp E. Bartko, Georg Goliasch, Christian Hengstenberg, Julia Mascherbauer

**Affiliations:** 1 Department of Internal Medicine II, Division of Cardiology, Medical University of Vienna, Waehringer Guertel 18-20, 1090 Vienna, Austria; matthias.koschutnik@meduniwien.ac.at (M.K.); varius.dannenberg@meduniwien.ac.at (V.D.); carolina.dona@meduniwien.ac.at (C.D.); christian.nitsche@meduniwien.ac.at (C.N.); andreas.kammerlander@meduniwien.ac.at (A.A.K.); sophia.koschatko@meduniwien.ac.at (S.K.); martin.huelsmann@meduniwien.ac.at (M.H.); stefan.aschauer@meduniwien.ac.at (S.A.); matthias.schneider@meduniwien.ac.at (M.S.); philippemanuel.bartko@meduniwien.ac.at (P.E.B.); georg.goliasch@meduniwien.ac.at (G.G.); christian.hengstenberg@meduniwien.ac.at (C.H.); 2 Department of Cardiac Surgery, Medical University of Vienna, Waehringer Guertel 18-20, 1090 Vienna, Austria; daniel.zimpfer@meduniwien.ac.at; 3 Department of Internal Medicine, Franziskus Hospital Margareten, Nikolsdorfergasse 32, 1050 Vienna, Austria; 4 Department of Internal Medicine and Cardiology, Charité-Universitätsmedizin Berlin (Campus Virchow-Klinikum), Augustenburger Platz 1, 13353 Berlin, Germany; 5 Department of Internal Medicine 3, University Hospital St. Poelten, Karl Landsteiner University of Health Sciences, Dunant-Platz 1, 3100 St. Poelten, Austria

**Keywords:** TMVR, surgical MV treatment, mitral valve, mitral regurgitation, outcome

## Abstract

Background. Transcatheter edge-to-edge mitral valve repair (TMVR) is increasingly performed. However, its efficacy in comparison with surgical MV treatment (SMV) is unknown. Methods. Consecutive patients with severe mitral regurgitation (MR) undergoing TMVR (68% functional, 32% degenerative) or SMV (9% functional, 91% degenerative) were enrolled. To account for differences in baseline characteristics, propensity score matching was performed, including age, EuroSCORE-II, left ventricular ejection fraction, and NT-proBNP. A composite of heart failure (HF) hospitalization/death served as primary endpoint. Kaplan-Meier curves and Cox-regression analyses were used to investigate associations between baseline, imaging, and procedural parameters and outcome. Results. Between July 2017 and April 2020, 245 patients were enrolled, of whom 102 patients could be adequately matched (73 y/o, 61% females, EuroSCORE-II: 5.7%, *p* > 0.05 for all). Despite matching, TMVR patients had more co-morbidities at baseline (higher rates of prior myocardial infarction, coronary revascularization, pacemakers/defibrillators, and diabetes mellitus, *p* < 0.009 for all). Patients were followed for 28.3 ± 27.2 months, during which 27 events (17 deaths, 10 HF hospitalizations) occurred. Postprocedural MR reduction (MR grade <2: TMVR vs. SMV: 88% vs. 94%, *p* = 0.487) and freedom from HF hospitalization/death (log-rank: *p* = 0.811) were similar at 2 years. On multivariable Cox analysis, EuroSCORE-II (adj.HR 1.07 [95%CI: 1.00–1.13], *p* = 0.027) and residual MR (adj.HR 1.85 [95%CI: 1.17–2.92], *p* = 0.009) remained significantly associated with outcome. Conclusions. In this propensity-matched, all-comers cohort, two-year outcomes after TMVR versus SMV were similar. Given the reported favorable long-term durability of TMVR, the interventional approach emerges as a valuable alternative for a substantial number of patients with functional and degenerative MR.

## 1. Introduction

Patients with severe mitral regurgitation (MR) suffer an annual mortality rate of 5% or more if left untreated [1]. Surgical treatment of MR (SMV) represents the second most common indication for valve surgery [2] and is recommended in symptomatic patients with acceptable operative risk. Valve repair remains the preferred surgical approach, if feasible [3,4]. Recently, transcatheter edge-to-edge mitral valve repair (TMVR) has emerged as a treatment alternative in patients with severe MR, who are considered not suitable for conventional heart surgery [5,6]. Based on emerging evidence of favorable long-term durability and outcome [7,8,9], TMVR is increasingly used in clinical practice [10,11,12].

Nevertheless, limited data on clinical performance, procedural efficacy, and outcome of TMVR compared with SMV are currently available [13,14,15]. The Endovascular Valve Edge-to-edge Repair Study (EVEREST II) [16] showed similar mortality at 5 years in TMVR and SMV patients (27 vs. 21%, *p* = 0.36), but higher re-operation rates, particularly within the first 6 months in patients with significant residual MR after TMVR (28 vs. 9%, *p* = 0.003). By today’s standards, EVEREST II can be seen as historical data, as technical improvements of the edge-to-edge repair technique and available devices have greatly improved procedural success rates [17,18]. According to recent guideline recommendations, TMVR is no more only considered for patients with functional MR and poor left ventricular (LV) systolic function [4,9], but has received a IIa recommendation in degenerative as well as functional MR in the newly issued ACC/AHA guidelines [4]. However, the indication upgrade for degenerative MR is based on registry data [8], and evidence comparing both techniques is scarce. Furthermore, new devices for TMVR have entered the market recently in order to target a wider spectrum of complex anatomical variations of the MV [17,19].

The aim of the present study was to provide a head-to-head comparison of TMVR versus SMV in a prospective, real-world, all-comers propensity score-matched cohort, focusing on procedural success and overall survival at two years.

## 2. Methods

### 2.1. Study Design

This observational study was performed at the Medical University of Vienna, a university-affiliated tertiary care center. Between July 2017 and April 2020, consecutive patients with severe MR, scheduled for TMVR and SMV, were prospectively recruited and retrospectively analyzed. Written informed consent was obtained in all patients prior to study enrollment. All cases were discussed by a multidisciplinary Heart Team. The study protocol was approved by our Institutional Review Board.

### 2.2. Echocardiography

Comprehensive echocardiographic assessments, including transesophageal echocardiography (TEE) in TMVR cases, were performed by board certified cardiologists using high-end scanners (Vivid 7, E9, E95, GE Healthcare; and EPIQ 7, Philips Medical Systems). Standard 2D and color-flow Doppler parasternal and apical views were analyzed. Cardiac chamber size was assessed according to current recommendations [20]. LV ejection fraction (LVEF) was calculated using the biplane Simpson’s method. Right ventricular (RV) systolic function was assessed using tricuspid annular plane systolic excursion (TAPSE) [21]. Systolic pulmonary artery pressure (PAP) was calculated by adding the peak tricuspid regurgitation (TR) systolic gradient to the estimated central venous pressure. Continuous-wave Doppler of the MV inflow was reviewed. Valvular heart disease was quantified using an integrated approach, as recommended in the respective guidelines [22,23]. Severity of MR was determined using morphological criteria and jet direction (myxomatous degeneration, leaflet prolapse/flail), as well as quantification by vena contracta width, estimated regurgitant volume, and the effective regurgitant orifice area. In accordance with the previously published literature [5,22], we applied a scale ranging from 1 to 4 in order to define MR severity: grade 1 indicates “mild”, 2 “moderate”, 3 “moderate-to-severe”, and 4 refers to “severe” MR. Readers of postprocedural echocardiographic exams did not belong to the interventional team and were blinded to procedural data, such as TEE performed intraoperatively, and outcome.

### 2.3. Mitral Valve Procedures

All TMVR procedures were performed under general anesthesia with TEE and fluoroscopic guidance [5,24]. In brief, the edge-to-edge mitral repair system was introduced through the femoral vein and advanced to the MV by crossing the inter-atrial septum. Up to three edge-to-edge devices were placed into the MV to maximally reduce MR. Surgical techniques included either MV repair with annuloplasty and/or chordae tendineae replacement, or MV replacement. The first operator case load for SMV and TMVR was, on average, 30 and 45 cases per year, respectively. 

### 2.4. Outcome Measures

Patients were prospectively followed in a dedicated outpatient clinic at 3 months, 12 months, and yearly thereafter. The primary outcome measure was a composite endpoint consisting of heart failure (HF) hospitalization and death. All-cause mortality was chosen as a secondary endpoint. Corresponding to recent data [25], we stratified patients according to the primary endpoint. Those who did not reach it at any time point were labeled “Super Responders”. Endpoints were ascertained by follow-up visits, state-wide electronic hospital charts, and patient phone calls. Mortality data were obtained via the National Death Registry (Statistics Austria).

### 2.5. Statistical Analysis and Propensity Matching

**Figure 1:**
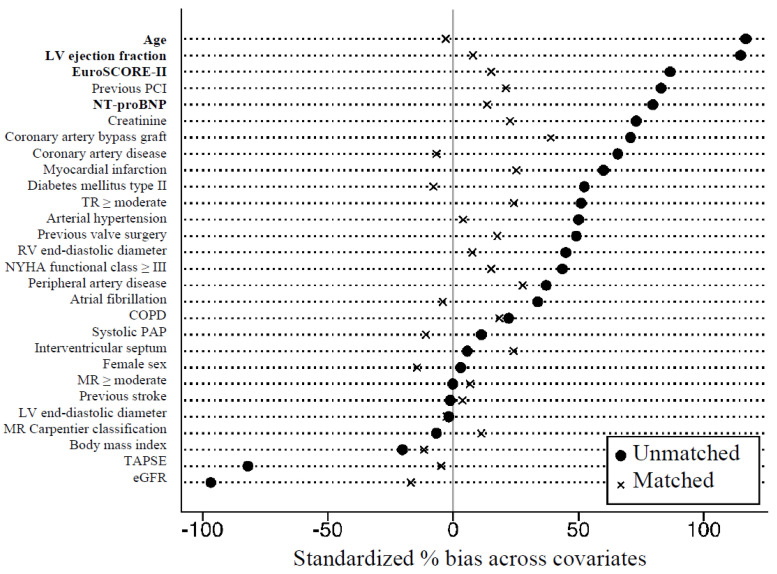
Standardized mean differences across covariates before (*n* = 245) and after propensity score matching (*n* = 102). Abbreviations: LV, left ventricular; NT-proBNP, N-terminal prohormone of brain natriuretic peptide; PCI, percutaneous coronary intervention; TR, tricuspid regurgitation; RV, right ventricular; NYHA, New York Heart Association; COPD, chronic obstructive pulmonary disease; PAP, pulmonary artery pressure; MR, mitral regurgitation; TAPSE, tricuspid annular plane systolic excursion; eGFR, estimated glomerular filtration rate.

Continuous data are presented as mean ± standard deviation (SD), with categorical variables being represented as total numbers and percentages. Comparisons between patient baseline characteristics and treatment groups were performed using either Chi-squared or Fisher’s exact tests for categorical variables or Wilcoxon rank-sum tests for continuous variables, as appropriate. Propensity score matching was performed according to the recommendations proposed by McMurry et al. [26]. A non-parsimonious multivariable logistic regression model was used to calculate propensity scores. Adjustment for significant differences in the patients’ baseline characteristics relevant for the treatment assignment and potential outcomes was performed with 1:1 matching using the following algorithm: nearest neighbor matching with a caliper width of 0.1 standard deviation of the propensity score and no replacement. The propensity score model was adjusted for differences in the following baseline characteristics: age, EuroSCORE-II, LVEF, and serum NT-proBNP levels. Figure 1 shows standardized mean differences across covariates before and after propensity score matching. Kaplan-Meier curves were plotted and the log-rank test was used to estimate differences between survival curves. Cox regression models were used to investigate associations between all baseline, imaging, and procedural parameters and the composite endpoint of HF hospitalization and death. In addition to crude analyses, we calculated multivariable models for both matched and unmatched study cohorts separately, which were adjusted for all parameters with a significant influence on a univariable level (EuroSCORE-II, NT-proBNP, coronary artery disease, atrial fibrillation, TAPSE, type of procedure, and MR postprocedural). Given the limited sample size, parameters already incorporated into the EuroSCORE-II were excluded from multivariable analyses. A two-sided *p*-value < 0.05 was considered statistically significant. All analyses were performed using SPSS 26 (IBM SPSS, Chicago, IL, USA) and Stata 15.1 (StataCorp, College Station, TX, USA).

## 3. Results

### 3.1. Baseline Characteristics

In total, 245 consecutive patients (103 TMVR, 142 SMV) were included between July 2017 and April 2020. After 1:1 propensity score matching, 102 (42%) entered the final analysis (51 TMVR, 51 SMV, Graphical Abstract). Supplementary Tables S1 and S2 demonstrate baseline characteristics and imaging data of the total study cohort.

Baseline characteristics of the matched study population are summarized in Table 1. Patients (72.5 ± 9.7 y/o, 61% females, EuroSCORE-II: 5.7 ± 5.7%, *p* > 0.05 for all) presented with advanced symptoms of HF (NYHA ≥III: TMVR 86 vs. SMV 77%, *p* = 0.255), NT-proBNP (TMVR: 3608 vs. SMV: 2192 pg/mL, *p* = 0.535), and renal function (estimated glomerular filtration rate (eGFR): TMVR: 59 vs. SMV: 69 mL/min/1.73 m^2^, *p* = 0.068). Despite matching, several baseline characteristics remained significantly different between TMVR and SMV patients. Prior myocardial infarctions were more frequent in TMVR (24 vs. 4%, *p* = 0.008), as well as prior percutaneous coronary interventions (33 vs. 4%, *p* < 0.001) and/or prior coronary artery bypass grafting (18 vs. 0%, *p* = 0.003). 24% of TMVR versus 4% of SMV patients carried pacemakers or defibrillators (*p* = 0.008). Diabetes mellitus was more prevalent in TMVR (33 vs. 12%, *p* = 0.009) as well as hyperlipidemia (75 vs. 45%, *p* = 0.002). Patients undergoing TMVR were on more advanced HF medication as reflected by more frequent use of sacubitril/valsartan (16 vs. 0%, *p* = 0.006), spironolactone (59 vs. 35%, *p* = 0.017), and loop diuretics (65 vs. 49%, *p* = 0.110).

Imaging data are shown in Table 2. Parameters were well balanced between propensity-matched groups (*p* > 0.065 for all). However, functional MR was more frequent among patients undergoing TMVR (61 vs. 16%, *p* < 0.001).

### 3.2. Procedural Data

Table 2 summarizes procedural data. Among surgical candidates, 67% underwent MV repair and 33% underwent MV replacement (Medtronic Mosaic: 71% (*n* = 12), Edwards Magna Ease: 24% (*n* = 4), On-X Mitral: 6% (*n* = 1)). Minimally invasive surgery via an intercostal approach was performed in 5 (10%) patients. A total of 27 (53%) patients in the SMV group underwent concomitant tricuspid valve procedures, whereas only 8 (16%) patients received transcatheter edge-to-edge tricuspid valve repair at the time of TMVR. Postprocedural MR reduction (MR grade < 2: 88 vs. 94%, *p* = 0.487) and MV mean pressure gradients (4.2 vs. 5.0 mmHg, *p* = 0.113) were similar for TMVR and SMV. Re-intervention/surgery rates for significant MV dysfunction were 6% for TMVR and 2% for SMV.

### 3.3. Cardiovascular Outcomes

**Figure 2:**
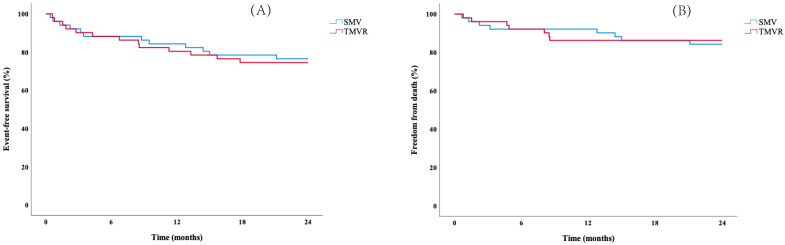
Kaplan-Meier curves stratified for type of intervention (surgical mitral valve treatment: SMV, transcatheter mitral valve repair: TMVR) regarding (**A**) the primary composite endpoint (heart failure hospitalization/death), and (**B**) all-cause death in the matched study population (*n* = 102).

**Figure 3:**
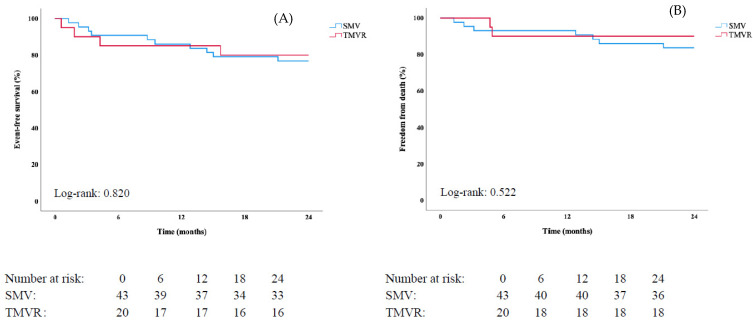
Kaplan-Meier curves stratified for type of intervention (surgical mitral valve treatment: SMV, transcatheter mitral valve repair: TMVR) regarding (**A**) the primary composite endpoint (heart failure hospitalization/death), and (**B**) all-cause death in patients with degenerative mitral regurgitation (MR) in the matched cohort (*n* = 63).

A total of 27 events (17 deaths, 10 HF hospitalizations) occurred during follow-up (mean 28.3 ± 27.2 months). Rates for HF hospitalization/death at 6 months, 1 year, and 2 years for TMVR were 12%, 20%, and 26%; and 12%, 16%, and 24% for SMV, respectively (log-rank: *p* = 0.811, Figure 2, Panel A). Similarly, no difference for overall survival at 2 years was found (TMVR vs. SMV: 86 vs. 84%, log-rank: *p* = 0.804, Figure 2, Panel B). In the degenerative MR cohort, 16 events (11 deaths, 5 HF hospitalizations) were observed. Log-rank tests showed no differences in outcome between TMVR and SMV (HF hospitalization/death: log-rank: *p* = 0.820, overall survival: log-rank: *p* = 0.522, Figure 3). Supplementary Figures S1 and S2 demonstrate differences between TMVR and SMV and endpoints in the unmatched study population.

Table 1 and Table 2 display baseline characteristics as well as imaging and procedural data stratified for “Super Responders” (*n* = 75, 72.4 ± 9.9 y/o, 56% females, EuroSCORE-II: 5.0 ± 3.5%) vs. “Non-responders” (*p* > 0.099 for all). Baseline and imaging characteristics did not significantly differ between groups. However, baseline NT-proBNP serum levels were lower (2158 vs. 4960 pg/mL, *p* = 0.017), and postprocedural residual MR was less common among “Super Responders” (MR grade <2: 97 vs. 74%, *p* = 0.001).

Results of the multivariable Cox-regression in the matched cohort are shown in Table 3. EuroSCORE-II (adj.HR 1.07 [95%CI: 1.00–1.13], *p* = 0.027) and postprocedural residual MR (adj.HR 1.85 [95%CI: 1.17–2.92], *p* = 0.009) emerged as independent predictors of event-free survival in the matched study population. In a second step, multivariable Cox-regression analysis was repeated in the unmatched study population. Again, EuroSCORE-II, baseline NT-proBNP serum levels, and postprocedural residual MR remained significantly associated with the primary endpoint, but not type of procedure (Supplementary Table S3).

## 4. Discussion

In this prospective cohort of propensity score-matched MR patients (38% functional, 62% degenerative MR) who underwent either TMVR or SMV, we report three main findings: (1) both techniques showed equivalent event-free survival at two years; (2) patients who did not meet the composite endpoint (HF hospitalization/death), labeled as “Super Responders”, were characterized by lower baseline NT-proBNP serum levels and less residual MR; and finally, (3) only EuroSCORE-II and residual MR were independently associated with outcome.

Over the past decade, TMVR has emerged as an accepted treatment option for MR patients at high surgical risk [3,27]. The 2020 ACC/AHA guidelines for the management of valvular heart disease have recently upgraded TMVR for the treatment of both degenerative and functional MR by giving it a level IIa recommendation [4]. However, prospective studies providing a head-to-head comparison of TMVR and SMV in degenerative as well as functional MR are still sparse, and results are contradictory. While several authors showed no difference in overall survival up to 5 years [16,28,29], others reported superior outcome after SMV [13,30]. The only prospective randomized controlled trial in this respect was the EVEREST II trial, published in 2011 [5]. A total of 279 patients with severe MR (70% degenerative) were randomized to receive either TMVR or SMV. Mortality rates at 5 years were similar (*p* = 0.36). However, this trial has one major limitation, namely the early experience of operators with TMVR. Overall, 28% of TMVR patients had to undergo second-line MV surgery, particularly within the first 6 months of follow-up, due to insufficient results of the initial intervention. In contrast, re-intervention rates in contemporary cohorts—such as the present one—are as low as 6% or less [8,9].

Takagi et al. [29] pooled 6 small retrospective observational studies (between 50 and 192 patients) and EVEREST II to compare outcomes after TMVR and SMV. Survival up to 5 years was similar (*p* = 0.46), although the logistic EuroSCORE was higher among TMVR patients (*p* < 0.001). Even in the randomized EVEREST II trial, congestive HF was significantly more prevalent among TMVR patients. A more recent multicenter registry included 568 TMVR and 173 SMV patients to compare both techniques [28]. After propensity score matching, no significant difference in overall survival at 5 years was found (*p* = 0.277). This registry, however, was also retrospective in design and, consequently, lacked data on HF hospitalization. Furthermore, information on matching variables was not disclosed.

In contrast to the aforementioned reports, Külling and coauthors [30] reported better survival after SMV than TMVR at 4 years (*n* = 185, *p* < 0.001). It has to be noted that although the EuroSCORE-II was significantly higher among TMVR patients (TMVR: 6.6% vs. MV repair: 1.7% vs. MV replacement: 3.6%), matching was not attempted, and the study design was retrospective. In line with these data, Buzzatti and colleagues also showed superior survival in 35 SMV versus 25 TMVR patients [13], but the study solely included patients with degenerative MR and was, once again, retrospective in design.

Taken together, definite evidence and consensus whether TMVR performs equally well compared with SMV is still lacking. In particular, recent and prospective head-to-head comparisons between both techniques in consecutive all-comers are not available.

The present analysis has several strengths: prospectively collected data, propensity score matching, inclusion of degenerative MR, and the use of HF hospitalization as an endpoint. In the total unmatched cohort (*n* = 245), 68% of TMVR but only 9% of SMV patients suffered from functional MR. TMVR patients were significantly older and presented with more comorbidities at baseline. Even after propensity score matching, accounting for age, EuroSCORE-II, LVEF and serum NT-proBNP, several co-morbidities remained more prevalent among TMVR patients. These included previous myocardial infarction, coronary artery bypass grafting, percutaneous coronary intervention, previous pacemaker implantation, and diabetes mellitus. Nevertheless, TMVR achieved similar survival at two years.

### Limitations

All data were collected in a single center; therefore, a perspective bias cannot be precluded. However, our single-center setting allows for consistency throughout the study period, including echocardiographic scanning conditions and post-processing workflows. Although postprocedural echocardiographic exams were performed independently of the interventional team, studies were not assessed by a central core laboratory. Allocation to study cohorts was performed by our local Heart Team based on current guidelines and recommendations. We used advanced statistical methods to harmonize divergent subgroups. However, due to the study design, the presence of confounding variables cannot be excluded, as patients were not randomized. Our results are generally more susceptible to bias caused by propensity score matching, mainly due to the relatively small sample size and predefined selection of matching variables. In addition, patients who did not reach the combined endpoint at any time point were labeled “Super Responders”; however, a comprehensive quality of life assessment was not available in all patients and was therefore not included in the final analysis. Given the small cohort size, results of the multivariable Cox regression models should be considered hypothesis generating. In addition, the follow-up time was limited to two years. Nonetheless, we learned from COAPT [31] and EVEREST II [16] that TMVR results remain stable at least up to five years. Another limitation refers to the non-randomized nature of our study, which may account for the relatively high rate of MV replacement surgery, as patients whose complex valve morphology was not well suited for either TMVR or MV repair were still included. Randomized controlled studies, such as the upcoming MATTERHORN trial (unique identifier: NCT02371512), are highly anticipated to confirm or disprove the present study results.

## 5. Conclusions

In this prospective, all-comers propensity score-matched cohort, two-year outcomes of SMV and TMVR were equal in terms of HF hospitalization and death, as well as in isolated degenerative MR. Given the previously reported excellent long-term durability of TMVR, the interventional approach arises as an acceptable alternative in an increasing number of patients with functional as well as degenerative MR. However, further large prospective studies are needed to optimize patient selection, choice of device and implantation techniques to further improve long-term clinical outcomes.

## Figures and Tables

**Table 1:** Baseline characteristics of the matched study population and stratified for “Super Responders” vs. “Non-responders”.

	All Patients (*n* = 102)	SMV (*n* = 51)	TMVR (*n* = 51)	*p* Value	Super Responders (*n* = 75)	Non-Responders (*n* = 27)	*p* Value
**Clinical parameters**							
Age (years)	72.5 ± 9.7	71.1 ± 10.1	74.0 ± 9.0	0.123	72.4 ± 9.9	73.0 ± 9.1	0.937
Female sex, n (%)	62 (61)	29 (57)	33 (65)	0.417	42 (56)	20 (74)	0.099
Body mass index (kg/m^2^)	27.1 ± 5.0	27.9 ± 4.9	26.2 ± 4.8	0.149	27.2 ± 5.1	27.1 ± 4.9	0.850
EuroSCORE-II (%)	5.7 ± 5.7	5.0 ± 3.9	6.8 ± 5.7	0.241	5.0 ± 3.5	7.6 ± 9.2	0.203
NYHA functional class ≥ III, n (%)	83 (81)	39 (77)	44 (86)	0.255	61 (81)	19 (70)	0.235
NT-proBNP (pg/mL)	2900 ± 4442	2192 ± 2504	3608 ± 5707	0.535	2158 ± 2543	4960 ± 7228	**0.017**
Creatinine (mg/dL)	1.3 ± 0.7	1.2 ± 0.7	1.4 ± 0.7	0.137	1.32 ± 0.7	1.5 ± 0.8	0.094
eGFR (mL/min/1.73m^2^)	63.6 ± 28.9	68.6 ± 28.2	58.6 ± 27.0	0.068	66.2 ± 29.0	56.4 ± 23.7	0.144
**Co-morbidities**							
Coronary artery disease, n (%)	42 (41)	17 (33)	25 (49)	0.108	30 (40)	12 (44)	0.687
Myocardial infarction, n (%)	14 (14)	2 (4)	12 (24)	**0.008**	10 (13)	4 (15)	0.848
Percutaneous coronary intervention, n (%)	19 (19)	2 (4)	17 (33)	**<0.001**	12 (16)	7 (26)	0.256
CABG, n (%)	9 (9)	0 (0)	9 (18)	**0.003**	7 (9)	2 (7)	1.000
Previous valve surgery, n (%)	8 (8)	2 (4)	6 (12)	0.269	4 (5)	4 (15)	0.203
Previous pacemaker implantation, n (%)	14 (14)	2 (4)	12 (24)	**0.008**	9 (12)	5 (19)	0.399
Atrial fibrillation, n (%)	69 (68)	33 (65)	36 (71)	0.525	47 (63)	22 (82)	0.073
Arterial hypertension, n (%)	100 (98)	50 (98)	50 (98)	1.000	73 (97)	27 (100)	1.000
Diabetes mellitus type II, n (%)	23 (23)	6 (12)	17 (33)	**0.009**	17 (23)	6 (22)	0.962
Hyperlipidemia, n (%)	61 (60)	23 (45)	38 (75)	**0.002**	44 (59)	17 (63)	0.696
Previous stroke, n (%)	8 (8)	5 (10)	3 (6)	0.715	2 (3)	6 (22)	**0.004**
Cerebral artery disease, n (%)	9 (9)	3 (6)	6 (12)	0.487	4 (5)	5 (19)	0.053
Peripheral artery disease, n (%)	5 (5)	1 (2)	4 (8)	0.362	3 (4)	2 (7)	0.606
COPD, n (%)	23 (23)	9 (18)	14 (28)	0.236	14 (19)	9 (33)	0.118
**Concomitant medication**							
Beta blockers, n (%)	80 (78)	42 (82)	38 (75)	0.336	56 (75)	24 (89)	0.174
ACE inhibitors, n (%)	36 (35)	21 (41)	15 (29)	0.214	28 (37)	8 (30)	0.473
Angiotensin receptor blockers, n (%)	27 (27)	16 (31)	11 (22)	0.262	21 (28)	6 (22)	0.560
ARNIs, n (%)	8 (8)	0 (0)	8 (16)	**0.006**	7 (9)	1 (4)	0.678
Calcium channel blockers, n (%)	16 (16)	8 (16)	8 (16)	1.000	14 (19)	2 (7)	0.225
Loop diuretics, n (%) / daily dose (mg)	58 (57)/46 ± 27	25 (49)/44 ± 29	33 (65)/48 ± 26	0.110	39 (52)/44 ± 27	19 (70)/50 ± 27	0.098
Thiazide diuretics, n (%) / daily dose (mg)	26 (26)/21 ± 13	19 (37)/20 ± 12	7 (14)/24 ± 15	**0.006**	21 (28)/20 ± 13	5 (19)/26 ± 14	0.332
Spironolactone, n (%) / daily dose (mg)	48 (47)/45 ± 21	18 (35)/54 ± 20	30 (59)/40 ± 21	**0.017**	32 (43)/40 ± 21	16 (59)/53 ± 20	0.139
Oral anticoagulants, n (%)	41 (40)	11 (22)	30 (59)	**<0.001**	29 (39)	12 (44)	0.600
Vitamin-K-Antagonists, n (%)	24 (24)	19 (37)	5 (10)	**0.002**	16 (21)	8 (30)	0.384
Statins, n (%)	53 (52)	19 (37)	34 (67)	**0.003**	39 (52)	14 (52)	0.989

Values are given as mean ± standard deviation or n (%). Abbreviations: SMV, surgical mitral valve treatment; TMVR, transcatheter mitral valve repair; NYHA, New York Heart Association; NT-proBNP, N-terminal prohormone of brain natriuretic peptide; eGFR, estimated glomerular filtration rate; CABG, coronary artery bypass graft; COPD, chronic obstructive pulmonary disease; ACE, angiotensin converting enzyme; ARNI, angiotensin receptor neprilysin inhibitor.

**Table 2:** Baseline imaging and procedural data of the matched study population and stratified for “Super Responders” vs. “Non-responders”.

	All Patients (*n* = 102)	SMV (*n* = 51)	TMVR (*n* = 51)	*p* Value	Super Responders (*n* = 75)	Non-Responders (*n* = 27)	*p* Value
**Echocardiographic parameters**							
LV end-diastolic diameter (mm)	51.9 ± 9.8	51.7 ± 7.8	52.0 ± 11.5	0.914	51.9 ± 10.6	51.6 ± 7.5	0.686
RV end-diastolic diameter (mm)	36.5 ± 6.5	36.1 ± 6.5	36.9 ± 6.5	0.549	35.9 ± 6.3	38.4 ± 6.9	0.171
Interventricular septum (mm)	13.0 ± 2.1	13.1 ± 1.8	12.8 ± 2.4	0.536	12.8 ± 2.3	13.4 ± 1.5	**0.045**
Aorta ascendens (mm)	34.5 ± 4.2	34.2 ± 4.4	34.7 ± 4.0	0.600	34.7 ± 4.3	33.7 ± 3.8	0.573
LV ejection fraction	55.1 ± 15.2	57.9 ± 13.8	52.3 ± 16.1	0.065	54.9 ± 15.5	55.8 ± 14.6	0.644
LV ejection fraction < 50%	29 (32)	13 (28)	16 (36)	0.415	19 (26)	8 (30)	0.719
LV ejection fraction < 30%	11 (12)	3 (6)	8 (16)	0.116	8 (11)	2 (7)	1.000
Systolic PAP (mmHg)	57.7 ± 17.5	59.9 ± 20.2	55.5 ± 14.1	0.245	57.1 ± 18.2	59.2 ± 15.8	0.549
TAPSE (mm)	19.2 ± 5.5	19.9 ± 6.2	18.4 ± 4.6	0.177	19.6 ± 5.7	18.0 ± 4.7	0.283
MR ≥ moderate, n (%)	102 (100)	51 (100)	51 (100)	1.000	75 (100)	27 (100)	1.000
MR etiology				**<0.001**			0.755
Degenerative, n (%)	63 (62)	43 (84)	20 (39)		47 (63)	16 (59)	
Functional, n (%)	39 (38)	8 (16)	31 (61)		28 (37)	11 (41)	
Carpentier classification							
Type I, n (%)	28 (27)	5 (10)	23 (45)		22 (29)	6 (22)	
Type II, n (%)	52 (51)	33 (65)	19 (37)		41 (55)	11 (41)	
Type IIIa, n (%)	11 (11)	10 (20)	1 (2)		6 (8)	5 (19)	
Type IIIb, n (%)	11 (11)	3 (6)	8 (16)		6 (8)	5 (19)	
TR ≥ moderate, n (%)	51 (51)	23 (46)	28 (55)	0.371	40 (54)	11 (41)	0.236
**Procedural data**							
No. of clips implanted							
1 (%), 2 (%), or 3 (%)	N/A	N/A	(72), (23), (5)		(74), (24), (2)	(68), (21), (11)	
NTR (n), XTR (n), or PASCAL (n)	N/A	N/A	(46), (35), (5)		(27), (27), (5)	(19), (8), (0)	
Type of surgery							0.822
MV repair, n (%)	N/A	34 (67)	N/A		25/37 (68)	9/14 (64)	
MV replacement, n (%)	N/A	17 (33)	N/A		12/37 (32)	5/14 (36)	
Concomitant TV procedure, n (%)	35 (34)	27 (53)	8 (16)	**<0.001**	26 (35)	9 (33)	0.900
Concomitant AV procedure, n (%)	N/A	8 (16)	N/A		3/37 (8)	5/14 (36)	
Concomitant CABG, n (%)	N/A	11 (22)	N/A		7/37 (19)	3/14 (21)	
MR postprocedural < moderate, n (%)	93 (91)	48 (94)	45 (88)	0.487	73 (97)	20 (74)	**0.001**
MV meanPG postprocedural (mmHg)	4.5 ± 2.0	5.0 ± 2.6	4.2 ± 1.3	0.113	4.4 ± 2.0	5.0 ± 2.1	0.173
Re-intervention/surgery, n (%)	4 (4)	1 (2)	3 (6)	0.308	3 (4)	1 (4)	0.946

Values are given as mean ± standard deviation or n (%). Abbreviations: SMV, surgical mitral valve treatment; TMVR, transcatheter mitral valve repair; LV, left ventricular; RV, right ventricular; PAP, pulmonary artery pressure; TAPSE, tricuspid annular plane systolic excursion; MR, mitral regurgitation; TR, tricuspid regurgitation; TV, tricuspid valve; AV, aortic valve; CABG, coronary artery bypass graft; meanPG, mean pressure gradient.

**Table 3:** Cox-regression analyses regarding associations with the primary composite endpoint (heart failure hospitalization/death) in the matched study population (*n* = 102). Multivariable analysis was adjusted for all parameters with a significant influence at an univariable level (EuroSCORE-II, NT-proBNP, atrial fibrillation, MR postprocedural), excluding those already incorporated in the EuroSCORE-II.

	HR	95% CI	*p* Value	Adj. HR	95% CI	*p* Value
	Univariable Analysis			Multivariable Analysis		
**Clinical parameters**						
Age	1.02	0.98–1.06	0.409			
Female sex	0.53	0.22–1.25	0.145			
Body mass index	1.00	0.93–1.08	0.999			
EuroSCORE-II	1.08	1.03–1.13	**0.001**	1.07	1.00–1.13	**0.027**
NYHA functional class ≥ III	0.74	0.32–1.68	0.467			
NT-proBNP (logarithmized)	2.88	1.35–6.12	**0.006**	1.42	0.61–3.30	0.422
Creatinine	1.40	0.96–2.03	0.079			
eGFR	0.99	0.97–1.00	0.067			
**Co-morbidities**						
Coronary artery disease	1.31	0.61–2.82	0.486			
Myocardial infarction	1.46	0.50–4.27	0.494			
Percutaneous coronary intervention	2.17	0.90–5.23	0.086			
CABG	1.30	0.30–5.57	0.724			
Previous valve surgery	3.36	1.14–9.87	**0.028**			
Previous pacemaker implantation	2.12	0.78–5.66	0.140			
Atrial fibrillation	2.89	1.09–7.68	**0.033**	2.36	0.82–6.79	0.112
Diabetes mellitus type II	1.15	0.46–2.87	0.760			
Hyperlipidemia	1.43	0.65–3.16	0.379			
Previous stroke	4.87	1.96–12.11	**0.001**			
Cerebral artery disease	3.71	1.37–10.05	**0.010**			
Peripheral artery disease	2.37	0.55–10.20	0.245			
COPD	2.08	0.93–4.65	0.074			
**Echocardiographic parameters**						
LV end-diastolic diameter	0.99	0.95–1.04	0.739			
RV end-diastolic diameter	1.06	1.00–1.14	0.072			
Interventricular septum	1.11	0.93–1.33	0.255			
Aorta ascendens	0.96	0.86–1.08	0.479			
LV ejection fraction	1.00	0.97–1.02	0.934			
LV ejection fraction < 50%	1.28	0.56–2.94	0.559			
LV ejection fraction < 30%	0.81	0.19–3.44	0.779			
Systolic PAP	1.00	0.98–1.02	0.862			
TAPSE	0.95	0.88–1.02	0.948			
MR etiology	1.58	0.72–3.45	0.256			
TR ≥ moderate	0.77	0.36–1.66	0.499			
**Procedural data**						
Type of procedure	1.65	0.73–3.72	0.225			
Concomitant TV procedure	0.91	0.41–2.04	0.913			
MR postprocedural	2.28	1.46–3.56	**<0.001**	1.85	1.17–2.92	**0.009**
MV meanPG postprocedural	1.07	0.91–1.25	0.443			
Re-intervention/surgery	1.97	0.26–14.97	0.514			

Abbreviations: NT-proBNP, N-terminal prohormone of brain natriuretic peptide; MR, mitral regurgitation; HR, hazard ratio; CI, confidence interval; Adj., adjusted; NYHA, New York Heart Association; eGFR, estimated glomerular filtration rate; CABG, coronary artery bypass graft; COPD, chronic obstructive pulmonary disease; LV, left ventricular; RV, right ventricular; PAP, pulmonary artery pressure; TAPSE, tricuspid annular plane systolic excursion; TR, tricuspid regurgitation; TV, tricuspid valve; meanPG, mean pressure gradient.

## Data Availability

Data presented in this study are available upon request from the corresponding author.

## Supplementary Materials

The following supporting information can be downloaded at: https://www.mdpi.com/article/10.3390/jpm12010090/s1, Figure S1: Kaplan-Meier curves stratified for type of intervention (surgical mitral valve treatment: SMV, transcatheter mitral valve repair: TMVR) regarding (A) the primary composite endpoint (heart failure hospitalization/death), and (B) all-cause death in the unmatched study population (*n* = 245).; Figure S2: Kaplan-Meier curves stratified for type of intervention (surgical mitral valve treatment: SMV, transcatheter mitral valve repair: TMVR) regarding (A) the primary composite endpoint (heart failure hospitalization/death), and (B) all-cause death for degenerative mitral regurgitation (MR) in the unmatched study population (*n* = 159). Table S1: Baseline characteristics of the unmatched study population.; Table S2: Baseline imaging and procedural data of the unmatched study population.; Table S3: Cox-regression analyses regarding associations with the primary composite endpoint (heart failure hospitalization/death) in the unmatched study population (*n* = 245). Multivariable analysis was adjusted for all parameters with a significant influence at an univariable level (EuroSCORE-II, NT-proBNP, coronary artery disease, atrial fibrillation, TAPSE, type of procedure, MR postprocedural), excluding those which are already incorporated into the EuroSCORE-II.

## Abbreviations

HF	Heart failure
LVEF	Left ventricular ejection fraction
MR	Mitral regurgitation
NT-proBNP	N-terminal prohormone of brain natriuretic peptide
SMV	Surgical mitral valve treatment
TMVR	Transcatheter edge-to-edge mitral valve repair
