# Supplementary material for: Transcatheter Versus Surgical Valve Repair in Patients with Severe Mitral Regurgitation
Source: J Pers Med. 2022 Jan 11;12(1):90. doi: 10.3390/jpm12010090 (PMC8779938; doi:10.3390/jpm12010090)
Supplement: Supplementary file 1 [file jpm-12-00090-s001.zip › jpm-1473872-supplementary.pdf]

Supplemental Material

Supplemental Figure S1. Kaplan-Meier curves stratified for type of intervention (surgical mitral valve treatment: SMV, transcatheter mitral valve repair: TMVR) regarding **A** the primary composite endpoint (heart failure hospitalization/death), and **B** all-cause death in the unmatched study population (n=245).

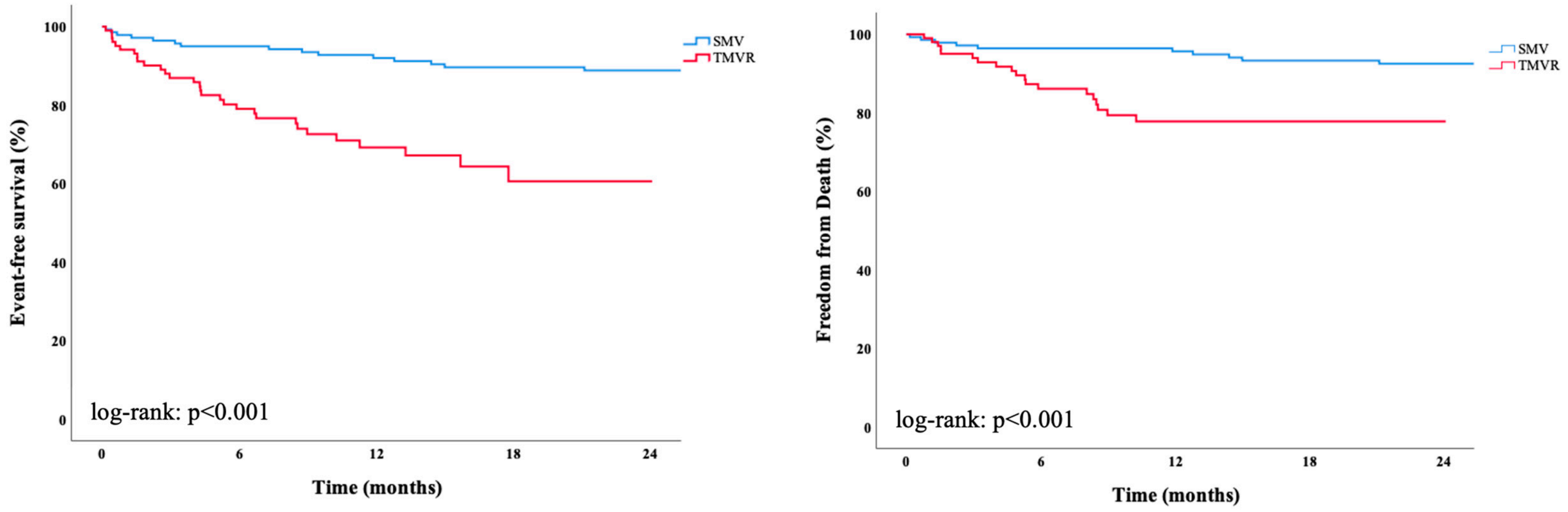

Number at risk:	0	6	12	18	24
SMV:	142	135	131	128	127
TMVR:	103	83	76	73	73

Number at risk:	0	6	12	18	24
SMV:	142	137	136	133	132
TMVR:	103	90	84	84	84

Supplemental Figure S2. Kaplan-Meier curves stratified for type of intervention (surgical mitral valve treatment: SMV, transcatheter mitral valve repair: TMVR) regarding **A** the primary composite endpoint (heart failure hospitalization/death), and **B** all-cause death for degenerative mitral regurgitation (MR) in the unmatched study population (n=159).

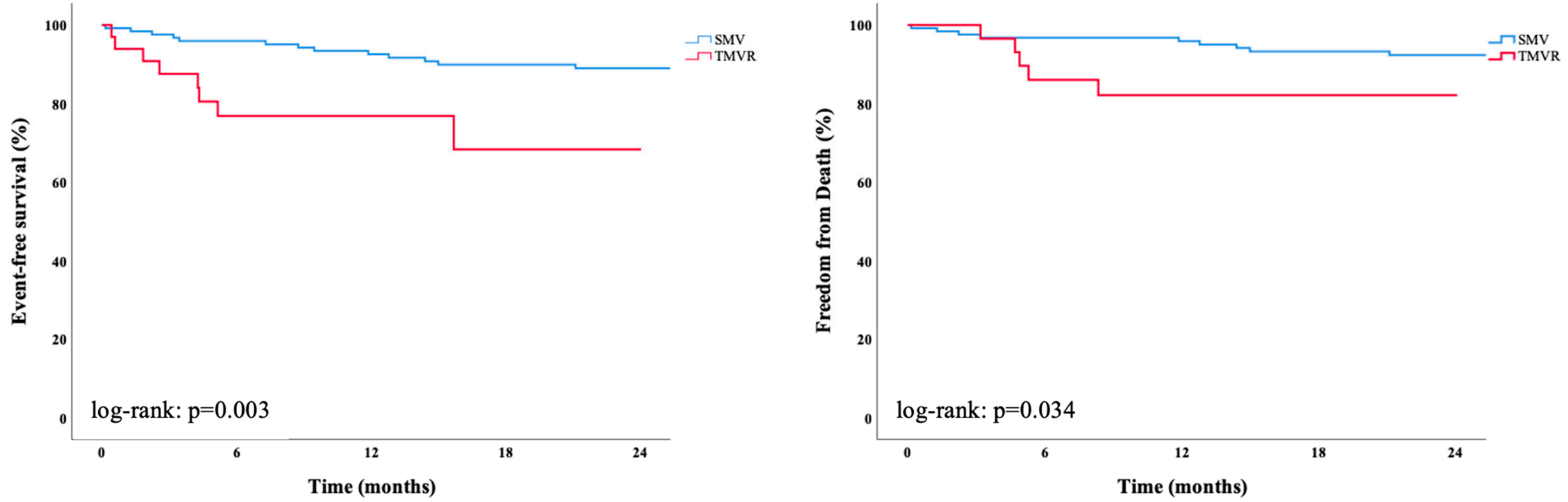

Number at risk:	0	6	12	18	24
SMV:	126	121	117	114	113
TMVR:	33	26	26	25	25

Number at risk:	0	6	12	18	24
SMV:	126	122	121	118	117
TMVR:	33	29	27	27	27

Supplemental Table S1. Baseline characteristics of the unmatched study population.

	All patients (n=245)	SMV (n=142)	TMVR (n=103)	*P* value
**Clinical parameters**				
Age (years)	69.7 ± 11.5	64.9 ± 10.9	76.2 ± 8.7	**<0.001**
Female sex, n (%)	147 (60)	87 (61)	60 (58)	0.692
Body mass index (kg/m^2)	26.6 ± 4.9	27.0 ± 5.0	26.0 ± 4.7	0.117
EuroSCORE-II (%)	5.9 ± 6.4	3.6 ± 2.8	8.9 ± 8.5	**<0.001**
NYHA functional class ≥ III, n (%)	190 (78)	103 (73)	87 (85)	**0.030**
NT-proBNP (pg/mL)	3555 ± 6248	1416 ± 1891	6357 ± 8486	**<0.001**
Creatinine (mg/dL)	1.3 ± 0.7	1.1 ± 0.5	1.6 ± 0.9	**<0.001**
eGFR (mL/min/1.73m^2)	63.3 ± 26.6	75.1 ± 23.5	51.6 ± 24.6	**<0.001**
**Co-morbidities**				
Coronary artery disease, n (%)	86 (35)	33 (23)	53 (52)	**<0.001**
Myocardial infarction, n (%)	29 (12)	5 (4)	24 (23)	**<0.001**
Percutaneous coronary intervention, n (%)	43 (18)	7 (5)	36 (35)	**<0.001**
Coronary artery bypass graft, n (%)	21 (9)	0 (0)	21 (20)	**<0.001**
Previous valve surgery, n (%)	19 (8)	3 (2)	16 (15)	**<0.001**

Previous pacemaker implantation, n (%)	40 (16)	3 (2)	37 (36)	**<0.001**
Atrial fibrillation, n (%)	133 (54)	67 (47)	66 (64)	**0.010**
Arterial hypertension, n (%)	223 (91)	122 (86)	101 (98)	**0.001**
Diabetes mellitus type II, n (%)	46 (19)	16 (11)	30 (29)	**<0.001**
Hyperlipidemia, n (%)	130 (53)	58 (41)	72 (70)	**<0.001**
Previous stroke, n (%)	20 (8)	12 (9)	8 (8)	0.847
Cerebral artery disease, n (%)	18 (7)	6 (4)	12 (12)	**0.028**
Peripheral artery disease, n (%)	13 (5)	2 (1)	11 (11)	**0.002**
COPD, n (%)	50 (20)	24 (17)	26 (26)	0.123
**Concomitant medication**				
Beta blockers, n (%)	177 (72)	94 (66)	83 (81)	**0.013**
ACE inhibitors, n (%)	70 (29)	41 (29)	29 (28)	0.902
Angiotensin receptor blockers, n (%)	61 (25)	38 (27)	23 (22)	0.429
ARNIs, n (%)	22 (9)	0 (0)	22 (21)	**<0.001**
Calcium channel blockers, n (%)	29 (12)	15 (11)	14 (14)	0.469
Loop diuretics, n (%) / daily dose (mg)	126 (51) / 52 ± 44	47 (33) / 44 ± 30	79 (77) / 48 ± 26	**<0.001**
Thiazide diuretics, n (%) / daily dose (mg)	42 (17) / 20 ± 14	30 (21) / 19 ± 14	12 (12) / 24 ± 15	0.052
Spironolactone, n (%) / daily dose (mg)	101 (41) / 49 ± 23	38 (27) / 55 ± 22	63 (61) / 40 ± 21	**<0.001**

Oral anticoagulants, n (%)	79 (32)	21 (15)	58 (56)	**<0.001**
Vitamin-K-Antagonists, n (%)	44 (18)	33 (23)	11 (11)	**0.011**
Statins, n (%)	107 (44)	46 (23)	61 (59)	**<0.001**

Values are given as mean ± standard deviation or n (%). Abbreviations: SMV, surgical mitral valve treatment; TMVR, transcatheter mitral valve repair; NYHA, New York Heart Association; NT-proBNP, N-terminal prohormone of brain natriuretic peptide; eGFR, estimated glomerular filtration rate; COPD, chronic obstructive pulmonary disease; ACE, angiotensin converting enzyme; ARNI, angiotensin receptor neprilysin inhibitor.

Supplemental Table S2. Baseline imaging and procedural data of the unmatched study population.

	**All patients (n=245)**	**SMV (n=142)**	**TMVR (n=103)**	***P* value**
**Echocardiographic parameters**				
LV end-diastolic diameter (mm)	52.9 ± 9.2	52.9 ± 7.8	52.9 ± 10.8	0.999
RV end-diastolic diameter (mm)	36.0 ± 7.0	34.6 ± 5.9	37.9 ± 7.9	**<0.001**
Interventricular septum (mm)	13.0 ± 2.3	13.0 ± 2.0	13.0 ± 2.7	0.970
Aorta ascendens (mm)	34.1 ± 4.3	33.9 ± 4.5	34.5 ± 4.0	0.316
LV ejection fraction	55.3 ± 15.7	61.8 ± 11.1	46.1 ± 16.8	**<0.001**
LV ejection fraction < 50%	68 (28)	16 (11)	52 (51)	**<0.001**

LV ejection fraction < 30%	27 (11)	3 (2)	24 (23)	**<0.001**
Systolic PAP (mmHg)	54.5 ± 17.2	53.5 ± 18.2	55.7 ± 15.9	0.344
TAPSE (mm)	19.8 ± 6.1	21.8 ± 6.5	17.1 ± 4.4	**<0.001**
MR ≥ moderate, n (%)	245 (100)	142 (100)	103 (100)	1.000
MR etiology				**<0.001**
Degenerative, n (%)	159 (66)	126 (91)	33 (32)	
Functional, n (%)	82 (34)	13 (9)	69 (68)	
TR ≥ moderate, n (%)	112 (46)	49 (35)	63 (62)	**<0.001**
**Procedural data**				
No. of clips implanted				
1 (%), 2 (%), or 3 (%)	N/A	N/A	(49.5), (47.6), (2.9)	
NTR (n), XTR (n), or PASCAL (n)	N/A	N/A	(76), (56), (8)	
Type of surgery				
MV repair, n (%)	N/A	108 (77)	N/A	
MV replacement, n (%)	N/A	33 (23)	N/A	
Concomitant TV procedure, n (%)	69 (28)	52 (37)	17 (17)	**0.001**

MR postprocedural < moderate, n (%)	226 (94)	132 (96)	94 (91)	0.096
MV meanPG postprocedural (mmHg)	4.3 ± 2.0	4.4 ± 2.4	4.2 ± 1.4	0.932

Values are given as mean ± standard deviation or n (%). Abbreviations: SMV, surgical mitral valve treatment; TMVR, transcatheter mitral valve repair; LV, left ventricular; RV, right ventricular; PAP, pulmonary artery pressure; TAPSE, tricuspid annular plane systolic excursion; MR, mitral regurgitation; TR, tricuspid regurgitation; TV, tricuspid valve; meanPG, mean pressure gradient.

Supplemental Table S3. Cox-regression analyses regarding associations with the primary composite endpoint (heart failure hospitalization/death) in the unmatched study population (n=245). Multivariable analysis was adjusted for all parameters with a significant influence at an univariable level (EuroSCORE-II, NT-proBNP, coronary artery disease, atrial fibrillation, TAPSE, type of procedure, MR postprocedural), excluding those which are already incorporated into the EuroSCORE-II.

	HR	95% CI	*P* value	Adj. HR	95% CI	*P* value
	Univariable analysis			Multivariable analysis		
**Clinical parameters**						
Age	1.07	1.03-1.10	**<0.001**			
Female sex	0.76	0.42-1.38	0.367			
Body mass index	1.00	0.95-1.06	0.986			
EuroSCORE-II	1.09	1.06-1.12	**<0.001**	1.05	1.01-1.09	**0.019**
NYHA functional class ≥ III	1.32	0.64-2.73	0.458			
NT-proBNP (logarithmized)	4.70	2.85-7.73	**<0.001**	2.50	1.33-4.68	**0.004**
Creatinine	1.63	1.32-2.00	**<0.001**			
eGFR	0.97	0.97-0.98	**<0.001**			
**Co-morbidities**						
Coronary artery disease	2.38	1.36-4.19	**0.003**	0.95	0.50-1.79	0.864

Myocardial infarction	2.10	1.01-4.37	**0.046**			
Percutaneous coronary intervention	2.49	1.33-4.67	**0.004**			
Coronary artery bypass graft	2.40	1.01-5.72	**0.047**			
Previous valve surgery	2.90	1.22-6.90	**0.016**			
Previous pacemaker implantation	3.95	2.13-7.34	**<0.001**			
Atrial fibrillation	3.57	1.82-7.00	**<0.001**	1.99	0.97-4.10	0.062
Diabetes mellitus type II	1.93	1.02-3.66	**0.043**			
Hyperlipidemia	1.48	0.83-2.62	0.182			
Previous stroke	2.66	1.25-5.68	**0.011**			
Cerebral artery disease	2.95	1.32-6.59	**0.008**			
Peripheral artery disease	3.31	1.26-8.15	**0.014**			
COPD	2.01	1.10-3.70	**0.024**			
**Echocardiographic parameters**						
LV end-diastolic diameter	1.00	0.97-1.03	0.879			
RV end-diastolic diameter	1.07	1.04-1.11	**<0.001**			
Interventricular septum	1.03	0.91-1.16	0.674			

Aorta ascendens	0.99	0.92-1.07	0.789			
LV ejection fraction	0.98	0.96-0.99	**0.003**			
LV ejection fraction < 50%	2.38	1.33-4.24	**0.003**			
LV ejection fraction < 30%	2.56	1.23-5.33	**0.012**			
Systolic PAP	1.01	1.00-1.03	0.113			
TAPSE	0.90	0.85-0.95	**<0.001**	0.99	0.93-1.06	0.799
MR etiology	3.04	1.71-5.43	**<0.001**			
TR ≥ moderate	1.64	0.93-2.88	0.088			
**Procedural data**						
Type of procedure	4.29	2.26-8.13	**<0.001**	1.13	0.53-2.42	0.745
Concomitant TV procedure	1.09	0.58-2.02	0.798			
MR postprocedural	2.75	2.01-3.76	**<0.001**	1.56	1.02-2.38	**0.040**
MV meanPG postprocedural	1.09	0.97-1.22	0.162			

Abbreviations: NT-proBNP, N-terminal prohormone of brain natriuretic peptide; TAPSE, tricuspid annular plane systolic excursion; MR, mitral regurgitation; HR, hazard ratio; CI, confidence interval; Adj., adjusted; NYHA, New York Heart Association; eGFR, estimated glomerular filtration rate; COPD, chronic obstructive pulmonary disease; LV, left ventricular; RV, right ventricular; PAP, pulmonary artery pressure; TR, tricuspid regurgitation; TV, tricuspid valve; meanPG, mean pressure gradient.
